# Consequences of Maternal Complications in Women's Lives in the First Postpartum Year: A Prospective Cohort Study

**DOI:** 10.3329/jhpn.v30i2.11318

**Published:** 2012-06

**Authors:** Kirti Iyengar, Ranjana Yadav, Swapnaleen Sen

**Affiliations:** ^1^Action Research and Training for Health, 772 Fatehpura, Udaipur 313001, India; ^2^Community Empowerment Laboratory, Lucknow, India

**Keywords:** Child survival, Cohort studies, Delivery, Delivery complications, Impact studies, Maternal health services, Maternal mortality, Pregnancy outcomes, Prospective studies, Rural health, India

## Abstract

Maternal complications are common during and following childbirth. However, little information is available on the psychological, social and economic consequences of maternal complications on women's lives, especially in a rural setting. A prospective cohort study was conducted in southern Rajasthan, India, among rural women who had a severe or less-severe, or no complication at the time of delivery or in the immediate postpartum period. In total, 1,542 women, representing 93% of all women who delivered in the field area over a 15-month period and were examined in the first week postpartum by nurse-midwives, were followed up to 12 months to record maternal and child survival. Of them, a subset of 430 women was followed up at 6-8 weeks and 12 months to capture data on the physical, psychological, social, or economic consequences. Women with severe maternal complications around the time of delivery and in the immediate postpartum period experienced an increased risk of mortality and morbidity in the first postpartum year: 2.8% of the women with severe complications died within one year compared to none with uncomplicated delivery. Women with severe complications also had higher rates of perinatal mortality [adjusted odds ratio (AOR)=3.98, confidence interval (CI) 1.96-8.1, p=0.000] and mortality of babies aged eight days to 12 months (AOR=3.14, CI 1.4-7.06, p=0.004). Compared to women in the uncomplicated group, women with severe complications were at a higher risk of depression at eight weeks and 12 months with perceived physical symptoms, had a greater difficulty in completing daily household work, and had important financial repercussions. The results suggest that women with severe complications at the time of delivery need to be provided regular follow-up services for their physical and psychological problems till about 12 months after childbirth. They also might benefit from financial support during several months in the postpartum period to prevent severe economic consequences. Further research is needed to identify an effective package of services for women in the first year after delivery.

## INTRODUCTION

Maternal conditions, such as haemorrhage, sepsis, and obstructed labour, as a group constitute one of the leading causes of the burden of disease for women of reproductive age throughout the world and contribute to high levels of mortality and disability in developing regions (1). According to the estimates of disability-adjusted life-years in the 1990 Global Burden of Diseases Study, reproductive ill-health accounts for 22% of the global burden of diseases among women of reproductive age (2). Maternal conditions dominate the burden of reproductive ill-health, accounting for 14.5% of the global burden of diseases, particularly in areas such as sub-Saharan Africa and India.

Despite the estimated large number of women who suffer from maternal complications, little is known about their health effects or the psychological, social and economic consequences (3). Most maternal health programmes have done little to reduce the burden of this consequent morbidity, which affects a large proportion of women. In part, this stems from our limited understanding of the incidence and prevalence of morbidity conditions, how these affect women's lives, how women and families respond to these, and how best to deal with these using a public-health approach.

The aim of this paper is to shed light on the physical, social, economic and psychological consequences of maternal complications occurring at the time of delivery or in the immediate postpartum period in the first year after childbirth. The specific objectives of the cohort study were to compare the physical, social, economic and psychological sequelae among women having severe and less-severe complications occurring around the time of delivery with those who had no such complications.

## MATERIALS AND METHODS

### Study design

We carried out a prospective cohort study in rural Rajasthan, a state of India, with high levels of maternal mortality (4), low levels of literacy, and widespread poverty (5). Southern Rajasthan is hilly, and villages are scattered across several hamlets. The study population comprised women residing in the field area of the Action Research and Training for Health (ARTH), a non-profit organization which covers a rural population of 58,000. The ARTH's field area has two health centres, located 25 and 52 km from Udaipur city, that offer 24-hour delivery and maternal and neonatal health services through 2-3 nurse-midwives posted at each centre (6). The ARTH's field programme tracks all pregnancies and all deliveries, irrespective of their place of delivery, through a community-based surveillance system.

### Recruitment of women for inclusion in the study

We selected women for the study from two sources:

- Women in their postpartum period from the community, who were detected to have a complication at the time of home-visits by the nurse-midwives: Trained nurse-midwives employed by the ARTH visited twice in the first week all women who delivered, irrespective of their place of delivery. They used a structured questionnaire-cum-examination form which included information on delivery-related complications, foetal outcomes, and symptoms and conducted a physical examination of mothers, including a haemoglobin test for anaemia (7). They also conducted a physical examination of babies, including weighing the babies. During May 2008–July 2009, 1,698 women delivered in the field area. Our nurse-midwives examined 1,542 women at their homes, representing 91% of the women who delivered during this period.
- Women presenting with maternal complications at ARTH's health centres were managed at these health centres or referred onward; 26% of cases were managed at ARTH's health centres during the study period. This information is recorded in “labour and maternal complication registers” maintained at the ARTH's health centres.

All postnatal forms, completed by the nurse-midwives during home-visit examinations for women who delivered during the reference period, were scrutinized by a social scientist between 4 and 6 weeks after delivery. The labour and maternal complication registers were also scrutinized. A morbidity-extraction checklist was used by the social scientists for classifying women into one of the three categories—severe maternal complication, less-severe maternal complication, and uncomplicated delivery.

### Definitions of severe maternal complications, less-severe maternal complications, uncomplicated delivery, and perinatal death

Our definition for severe complication was based on hospital management and postpartum examinations carried out by the nurse-midwives during postpartum home-visits. Table 1 shows the criteria for inclusion into these categories.

These conditions were mutually exclusive; for example, if a woman with severe anaemia received blood transfusion, we categorized her under “woman given blood transfusion for any reason in pregnancy or postpartum period.”

We hypothesized that the conditions mentioned under severe complications are life-threatening while less-severe complications are not immediately life-threatening but can cause disability to women. These definitions for severe complications are not the same as the definition used in other studies for ‘near-miss’ (8) as we selected women who delivered either at home or at a facility; if the woman delivered at home, the complication was first detected by a nurse-midwife through a structured questionnaire at the time of a postpartum visit in the first week after delivery.

### Sampling

Of the 1,698 women who delivered during the study period in our field area, 1,542 (90.8%) were contacted during the postpartum period and were assigned to one of the morbidity categories. Village health workers appointed by ARTH followed up all enrolled women up to 12 months after delivery to record the survival of mothers and infants. If a woman was not available, information was collected from a close family member regarding survival of mother and the baby. In other words, the sample included all 1,542 women and infants for the purpose of recording survival.

**Table 1.** Definitions of various categories of women who delivered

Women with ‘severe’ maternal complication
a. Eclampsia
b. Woman given blood transfusion for any reason in pregnancy or postpartum period
c. Severe postpartum haemorrhage who required blood transfusion
d. Severe anaemia (haemoglobin level 7 g or below) at postpartum visit in the first week
e. Complicated abortion that required laparotomy or blood transfusion
f. Caesarean section for maternal indication (antepartum haemorrhage, obstructed labour, transverse lie, and twin pregnancy)
Women with ‘less-severe’ maternal complications
a. Severe and moderate pre-eclampsia
b. Postpartum haemorrhage as noticed by a skilled birth attendant but no blood transfusion given
c. Antepartum haemorrhage (significant amount of blood loss before delivery as per records/reported by woman) but no caesarean section or blood transfusion done
d. Moderate anaemia (Hb 7.1-9.0 g) at postpartum visit in the first week
e. Abortion-related complication that required uterine evacuation
f. Caesarean section for any reason but not maternal indication
g. Fever >38 °C reported by nurse-midwife/doctor or infected episiotomy/sepsis
Uncomplicated group
a. Vaginal delivery without severe and less-severe maternal complications as mentioned above

A subset of these women were recruited at 6-8 weeks post-delivery for a detailed study on the psychological, physical, economic and social consequences. Since there were many outcomes of interest and the frequencies of many of which were not known, the sample-size was decided based on the best guess of the number of women that would reflect the 10% difference between those with severe complication and the uncomplicated group for most outcomes. Given their small numbers, an effort was made to sample all women who were classified as having experienced severe complications. Women who had less-severe complications or uncomplicated deliveries were recruited such that once a certain number of women in these categories had been interviewed (200 for less-severe and 200 normal births), no further efforts were made to recruit more such women. A few women in the list could not be contacted at the time of visit between six and eight weeks even after three visits; they were not included in the study. Our research investigators were provided with a list of all women with severe, less-severe and uncomplicated deliveries every month. When they went to a village to contact women with severe or less-severe complications, they also contacted women with uncomplicated births in the same village.

### Collection of data

A team of four research investigators collected data during June 2008–July 2010 by visiting these women at two time-points after delivery (at 6-8 weeks and 12 months). Of 430 women interviewed at 6-8 weeks, 375 (87.2%) could be successfully contacted at 12 months. The remaining women could not be contacted even after three visits at their houses. An attempt was made to include all women with severe complications.

### Developing the indicators and structured questionnaire

Before commencing the study, we had conducted a qualitative study using freelists and in-depth interviews with key-informants to determine the local terms used for describing morbidities and for understanding the various dimensions of the consequences. This helped us develop a conceptual framework for the research presented in this paper. Based on outcomes of interest and indicators, we developed a structured questionnaire in Hindi and Mewari, which covered questions relating to the physical, social, psychological and economic consequences of maternal complications and the care-seeking pattern. The draft questionnaire was pretested in the field and then finalized for use.

For assessing postpartum depression, the Edinburgh Postpartum Depression Scale (EPDS) was modified and adapted to the local situation with the help of a psychiatrist. It was developed into a two-point scale on 10 questions (see box). A positive response to three of the 10 questions was considered to be postpartum depression. A psychiatrist conducted a two-day training for the interviewers, which included both classroom sessions and field training.

Women were also asked about the annual household income in the first round of interviews (6-8 weeks). They were also asked about the expenditure incurred on delivery and postpartum treatment. Catastrophic expenditure was calculated at 10% or more of the annual income level.

### Statistical analysis

Data were entered into the Epi Info software (version 6) and analyzed using the Stata software (version 11) to derive percentages, means, levels of significance, unadjusted and adjusted odds ratios.

### Ethical issues

The institutional ethics committee of the ARTH approved the study. The investigators obtained verbal consent of women for interview, since most women were illiterate. Women with complications recruited for the study were referred to the health centres by the research investigators and were offered free treatment.

## RESULTS

### Number of women covered and proportions with severe and less-severe complications

In total, 1,698 women delivered during May 2008–July 2009 in our field area, of whom 1,542 (90.8%) could be contacted during the early postpartum period and were categorized into one of the three morbidity categories. Of them, 7.4% had one or more severe complication(s), 36.5% had a less-severe complication, and 56.1% had an uncomplicated delivery (Table 2).

For studying the survival of mothers and babies, we present data from all women who delivered in the field area during the study period; they were categorized into one of the three morbidity categories during postpartum visits.

Of 430 contacted at 6-8 weeks, 84 had been detected to have severe complications at delivery or the time of the nurse-midwife's visit in the week following delivery, 162 had less-severe complications, and 184 had uncomplicated deliveries. At 12 months, 46 women could not be contacted despite three visits to their homes, six women refused interview, and three had died. Hence, 375 women (87.2%) could be interviewed at 12 months (Table 2). The proportions of recruited women who were followed up to 12 months (responders) included: 90% (n=165) for uncomplicated deliveries, 86% (n=139) for less-severe complications, and 85% (n=71) for severe complications.

**Table 2.** Numbers and percentages of women recruited by morbidity category: severe complication, less-severe complication, and uncomplicated delivery

Number of women	Severe maternal complications	Less-severe complications	Uncomplicated deliveries	Total
No. of women who delivered during May 2008–July 2009	110	572	860	1,542
% of delivering women in different categories	7.4	36.5	56.1	100
No. of women who attended interview at 6-8 weeks	84	162	184	430
Number of women approached for interview at 12 months	84	162	184	430
• Lost to follow-up	9	22	15	46
• Refused	2	1	3	6
• Died	2	0	1	3
• Attended interview at 12 months	71	139	165	375

### Type of morbidity at time of delivery or immediately postpartum

More than two-thirds of the 84 women with severe complications were those with severe anaemia while another 17% were those who had received blood transfusion for any reason during the pregnancy or postpartum period (Table 3). The remaining women were those with caesarean section for absolute maternal indication or those who had eclampsia. Most (80.9%) women with less-severe complications were those with moderate anaemia (59.3%) or those who had suffered postpartum haemorrhage but had not received a blood transfusion (21.6%).

**Table 3.** Types of complications among women with severe and less-severe complications

Type of complication	Severe complication N (%)	Less-severe complication N (%)
Eclampsia	3 (3.6)	NA
Severe PPH requiring blood transfusion	4 (4.8)	NA
Women given blood transfusion for other reason in maternal period	10 (11.9)	NA
Severe anaemia (haemoglobin 7 g or less)	60 (71.4)	NA
Caesarean section for absolute maternal indication	7 (8.3)	NA
Severe/moderate pre-eclampsia	NA	11 (6.8)
PPH as noticed by a skilled birth attendant but no blood transfusion	NA	35 (21.6)
Antepartum haemorrhage but no CS or blood transfusion done	NA	5 (3.1)
Moderate anaemia (haemoglobin 7.1-9 g or less)	NA	96 (59.3)
Abortion-related complication requiring uterine evacuation	NA	2 (1.2)
CS for non-maternal indications	NA	4 (2.5)
Puerperal sepsis/infected episiotomy	NA	9 (5.6)

CS=Caesarean section; NA=Not applicable; PPH=Postpartum haemorrhage

### Profile of women

Thirty-eight percent of the 430 women delivered at home while the remaining delivered in institutions. More than two-thirds belonged to the socioeconomically-backward scheduled tribes or scheduled castes (Table 4). Less than 10% of the women were literate. Nearly 45% stayed in nuclear families while the remaining women stayed in joint families. Almost one-fifth of the women were primiparas. Only 2.5% had delivered by caesarean section.

The women recruited were similar across the morbidity categories in terms of place of delivery and previous number of children (Table 4). However, women in the severe category had lower levels of income and were more likely to belong to a scheduled caste or tribe and to have suffered perinatal death. These differences were not significant.

### Survival of mothers and infants

Among the 1,542 women who survived delivery and the first week postpartum during May 2008–July 2009, the 12-month follow-up data showed that there were a total of five deaths. Among women with severe complications, there were four deaths while one woman with uncomplicated delivery had an accidental death (Table 5). None of the women with less-severe complications had died. Among four deaths that occurred in the severe complication group, two women had maternal deaths, one had a late maternal death, and one had an accidental death. Verbal autopsies suggest that the causes of maternal deaths were severe anaemia and secondary PPH while the late maternal death occurred due to severe anaemia and infection (Table 5). Of the three women with severe complications who had maternal or late maternal deaths, one each delivered at home, government subcentre, and a district-level government hospital.

Data on the survival of babies during the study period revealed that, compared to women with uncomplicated delivery, women with severe or less-severe maternal complications were nearly 3.3 times and 2.3 times more likely to have had perinatal deaths respectively. This difference was highly significant (Table 6) for both severe (p<0.0001) and less-severe (p<0.0001) complication groups.

Even after excluding the perinatal period, women with severe complications were 2.7 times more likely to lose their babies between eight days and 12 months after delivery (p=0.004) (Table 6). The rate of mortality of babies between eight days and 12 months was 9.4% compared to 5% in the less-severe complication group and 3.4% in the uncomplicated group. Even normal-weight babies (2.5 kg or higher weight during postpartum visit) born to women with severe complications were three times as likely to die between eight days and 12 months than normal weight babies born to women without complications (Table 5). Low-birthweight babies born to mothers with severe complications were 2.2 times more likely to die between eight days and 12 months.

**Table 4.** Background characteristics of women with complications or normal delivery

Profile of women	Severe complications (n=84)	Less-severe complications (n=162)	Normal (n=184)	Total (n=430)
Caste, no. (%)				
Scheduled caste/tribe	62 (73.8)	104 (65.6)	122 (69.7)	234 (63.9)
Others	22 (25.7)	58 (34.4)	62 (30.3)	132 (36.1)
Husband's annual income in Indian rupees (US$)				
Mean	24,263 (516)	29,457 (627)	25,223 (537)	26,677 (568)
Median	21,700 (462)	24,500 (521)	24,000 (511)	24,000 (511)
Range	6,000-60,000 (128-1,277)	2,400-120,000 (51-2,553)	6,000-96,000 (128-2,043)	2,400-120,000 (51-2,553)
Type of family, no. (%)				
Nuclear	41 (48.8)	67 (41.4)	83 (45.1)	191 (44.4)
Joint	43 (51.2)	95 (58.6)	101 (54.9)	239 (55.6)
Place of delivery, no. (%)				
Home	27 (38.6)	44 (33.6)	67 (40.6)	138 (37.7)
Institutional				
ARTH's health centres	18 (25.7)	38 (29.0)	41 (24.8)	97 (26.5)
Government subcentre/primary health centre/community health centre	4 (5.7)	20 (15.3)	28 (17.0)	29 (7.9)
Government district hospital	21 (30.0)	26 (19.8)	22 (13.3)	69 (18.9)
Private and other facilities	0	3 (2.3)	7 (4.2)	10 (2.7)
No. (%) of children ever born				
1	19 (22.6)	35 (21.6)	43 (21.6)	97 (22.6)
2-3	36 (42.9)	70 (45.8)	74 (43.2)	180 (41.9)
4-5	21 (25.0)	42 (25.9)	51 (25.9)	114 (26.5)
6+	8 (9.5)	15 (9.3)	15 (9.3)	39 (9.1)
Caesarean section	7 (8.3)	4 (2.5)	0	11 (2.5)

**Table 5.** Survival of mothers and babies up to 12 months postpartum

Outcome	Severe (n=110)	Less severe (n=572)	Normal (n=860)	Total (n=1,542)
Maternal outcome				
Maternal deaths	2	0	0	2
Late maternal deaths	1	0	0	1
Accidental deaths	1	0	1	2
% of mothers who suffered maternal death or late maternal death	2.8	0	0	0.2
Perinatal outcome				
Number of perinatal deaths (stillbirths + early neonatal deaths)	13	47	31	91
Perinatal mortality rate	118.2	82.2	36.0	59.0
Outcome of babies between 8 days and 12 months				
Deaths of babies between 8 days and 12 months after birth	10	28	29	67
Mortality rate among babies aged 8 days to 12 months (per 1,000 livebirths)	93.5	51.1	34.2	44.5
For low-birthweight babies (<2.5 kg)	135.1	67.1	61.4	70.0
For babies having normal weight (2.5 kg and above)	67.8	47.4	23.1	34.4

**Table 6.** Maternal and child survival: levels of statistical significance and odds ratios of severe and less-severe complication groups compared to those with uncomplicated group at 12 months

Outcome	Compared to women with uncomplicated group	
	Severe complications	Less-severe complications
Maternal survival	p<0.0001 OR undefined	OR undefined
Perinatal mortality	p=0.0001 OR=3.583 (1.81-7.08) AOR=3.986 (1.96-8.1)	p=0.0001 OR=2.39 (1.5-3.81) AOR=2.57 (1.59-4.13)
Child mortality between 8 days and 12 months	p=0.004 OR=3.242 (1.46-7.2) AOR=3.14 (1.4-7.06)	p=0.481 OR=1.25 (0.67-2.31) AOR=1.21 (0.64-2.26)

AOR=Adjusted odds ratio; OR=Odds ratio

### Psychological consequences

Women with severe complications were significantly more at risk of depression at 6-8 weeks after delivery than women with uncomplicated births (Table 7 and 9) (p=0.001). However, these differences were reduced at 12 months. At 6-8 weeks, 2.2% of women with uncomplicated births and 4.8% with severe complications had suicidal thoughts but the difference was not significant. The prevalence of suicidal thoughts declined at 12 months postpartum (Table 7 and 9).

### Economic consequences

A greater proportion of women with severe complications received postpartum treatment compared to women with normal delivery both at 6-8 weeks and 12 months (p≤0.00001). At 6-8 weeks, women with severe complications were significantly more likely to have borrowed money to receive such treatment (AOR=5.90, p=0.001). One in six women with severe complications was indebted at 6-8 weeks compared to 2.2% in uncomplicated group (p<0.000), and 4.2% were still indebted at the time of interview at 12 months but the difference was not significant at 12 months (Table 8 and 9). Nearly 11% of women with severe complications had incurred catastrophic expenditure (defined here as more than 10% annual household income) for their treatment, and this was significantly higher than for those with uncomplicated delivery both at 6-8 weeks and 12 months (Table 8 and 9). Women with less-severe complications also incurred catastrophic expenditure more often than those with uncomplicated delivery.

Interestingly, at 6-8 weeks, more than half of all women reported that jobs of their husbands were affected after delivery. Compared to those with uncomplicated delivery, a higher proportion of women with severe complications reported that jobs of their husbands were affected, income of their husbands reduced, and that their financial condition had worsened after delivery. The differences were significant at 6-8 weeks but there was no significant difference between various categories at 12 months (Table 8 and 9). Most men worked in larger cities as seasonal migrants or daily-wagers and returned home when needed. Hence, they might have had to remain at home more at the time of delivery and then stayed longer with their wives if they suffered complications.

**Table 7.** Psychological consequences at 6-8 weeks and 12 months postpartum among women with severe complications, less-severe complications, and women with normal delivery

Psychological consequence	6-8 weeks postpartum			12 months postpartum		
	Severe (n=84) No. (%)	Less severe (n=162) No. (%)	Uncomplicated (n=184) No. (%)	Severe (n=71) No. (%)	Less severe (n=139) No. (%)	Uncompli-cated (n=165) No. (%)
Postpartum depression	28 (33.3)	30 (18.5)	29 (15.8)	8 (11.3)	13 (9.4)	11 (6.7)
Suicidal thoughts since delivery	4 (4.8)	2 (1.2)	4 (2.2)	2 (2.8)	4 (2.9)	6 (3.6)

**Table 8.** Care-seeking patterns and economic consequences of women with severe complications, less-severe complications, and normal delivery

Care-seeking patterns and economic consequences	6-8 weeks			12 months		
	Severe (n=84) No. (%)	Less severe (n=162) No. (%)	Uncomplicated (n=184) No. (%)	Severe (n=71) No. (%)	Less severe (n=139) No. (%)	Uncompli-cated (n=165) No. (%)
Postpartum treatment received	35 (41.7)	44 (27.2)	30 (16.3)	33 (46.5)	38 (27.3)	38 (23.0)
Borrowed or mortgaged to receive treatment	12 (14.3)	7 (4.3)	5 (2.7)	9 (12.7)	2 (1.4)	7 (4.2)
Indebted at the time of interview	13 (15.5)	6 (3.7)	4 (2.2)	3 (4.2)	1 (0.7)	4 (2.4)
Catastrophic expenditure (spent >10% of annual household income)	9 (10.7)	3 (1.9)	2 (1.1)	7 (9.9)	4 (2.9)	1 (0.6)
Husband's job affected due to delivery	60 (71.4)	94 (58.0)	100 (54.3)	21 (29.6)	48 (34.5)	57 (34.5)
Income reduced after delivery	53 (63.1)	79 (48.8)	78 (42.4)	20 (28.2)	45 (32.4)	54 (32.7)
Financial conditions have worsened after delivery	34 (40.5)	40 (24.7)	44 (23.9)	13 (18.3)	13 (9.4)	36 (21.8)

### Social consequences

While at 6-8 weeks, there were no significant differences in the ability to care for themselves, a significantly greater proportion of women with severe complications were not able to take care of themselves as before at 12 months (p=0.001, OR=5.38) (Table 10).

The most common difficulty was in resuming household work (defined as inability to do household work or doing it with difficulty). At 6-8 weeks, a greater proportion of women with severe and less-severe complications reported difficulty in resuming their household work (p=0.001 for both the groups). At 12 months, only 4% of women with normal delivery compared to 17% with severe complications continued to report difficulty in resuming household work (p=0.04).

Between 2 and 3% of women reportedly faced adverse behaviour from their spouses or families but the difference was not significant across the three groups. One in six women in the severe complication group did not resume sexual relationship because of illness at 6-8 weeks compared to 4% in the less-severe complication group and uncomplicated group (p=0.001). The difference was not significant at 12 months.

### Perception of health

Women with severe and less-severe complications reported various symptoms more frequently than women with uncomplicated delivery at 6-8 weeks. Women with severe complications were significantly more likely to report feeling unwell, fever, swelling of body, weakness, pain in arms and legs, and excessive bleeding (Table 11 and 12) at 6-8 weeks. Most of these symptoms continued till 12 months among women with severe complications. Interestingly, women with severe complications were also twice more likely to report having cough at 6-8 weeks and 12 months, although the differences were not significant at 12 months. There were no significant differences between the three groups of the women in terms of abdominal pain, vaginal discharge, urinary burning, and breast pain.

Symptoms indicative of serious illness (i.e. more than 3 of the following symptoms: fever, swelling, yellowness of body, feeling extremely weak, ache in hands and legs, excessive bleeding, breathlessness, cough, and abdominal pain) were also reported more commonly by women with severe complications compared to women with uncomplicated delivery (Table 11 and 12). Women with less-severe complications also reported several symptoms more frequently than women in the uncomplicated group; however, the differences were not significant.

**Table 9.** Psychological, social and economic consequences: levels of statistical significance and odds ratios of severe and less-severe complication group compared to those with uncomplicated group at 6-8 weeks and 12 months

Outcome	Compared to women with uncomplicated deliveries			
	Severe complications		Less-severe complications	
	6-8 weeks	12 months	6-8 weeks	12 months
Psychological consequences				
Depression	p=0.001 OR=2.6 (1.4-4.8) AOR=3.6 (1.6-5.7)	p=0.239 OR=1.71 (0.68-4.62) AOR=1.60 (0.58-4.39)	p=0.497 OR=1.2 (0.69-2.12) AOR=1.2 (0.67-2.1)	p=0.389 OR=1.44 (0.625-3.33) AOR=1.44 (0.614-3.402)
Suicidal thoughts	p=0.260 OR=2.25 (0.54-9.22) AOR=5.18 (0.90-29.7)	p=0.750 OR=0.768 (0.151-3.90) AOR=0.513 (0.08-3.19)	p=0.51 OR=0.56 (0.10-3.11) AOR=0.96 (0.14-6.29)	p=0.712 OR=0.785 (0.22-2.84) AOR=0.804 (0.22-2.95)
Social consequences				
Self-care less than before	p=0.646 OR=1.16 (0.61-2.19) AOR=1.29 (0.65-2.56)	p=0.001 OR=5.38 (1.93-15.0) AOR=6.92 (2.19-21.78)	p=0.232 OR=0.70 (0.396-1.25) AOR=0.73 (0.40-1.34)	p=0.048 OR=2.73 (1.01-7.39) AOR=3.45 (1.14-10.41)
Difficulty in resuming household work	p=0.001 OR=2.4 (1.43-4.22) AOR=2.8 (1.58-5.0)	p=0.000 OR=3.443 (1.928-6.150) AOR=3.596 (1.935-6.682)	p=0.093 OR=1.4 (0.94-2.20) AOR=1.48 (0.95-2.30)	p=0.0480 OR=1.19 (0.74-1.91) AOR=1.26 (0.76-2.08)
Suffered violence/verbal abuse/ignored	p=0.513 OR=0.64 (0.17-2.40) AOR=1.05 (0.23-4.64)	p=0.628 OR=1.56 (0.25-9.57) AOR=2.20 (0.29-16.41)	p=0.633 OR=0.785 (0.29-2.11) AOR=1.07 (0.32-3.5)	p=0.832 OR=1.19 (0.23-5.99) AOR=1.37 (0.26-7.23)
Not resumed sexual relations	p=0.851 OR=1.06 (0.53-2.14) AOR=1.00 (0.47-2.13)	p=0.390 OR=0.624 (0.213-1.82) AOR=0.614 (0.207-1.822)	p=0.266 OR=1.36 (0.78-2.37) AOR=1.36 (0.77-2.41)	p=0.280 OR=0.61 (0.25-1.49) AOR=0.63 (0.26-1.58)
Treatment-seeking and economic consequences				
Postpartum treatment received	p=0.000 OR=3.6 (2.04-6.57) AOR=2.9 (1.5-5.5)	p=0.000 OR=2.90 (1.60-5.23) AOR=2.83 (1.51-5.30)	p=0.015 OR=1.9 (1.13-3.22) AOR=1.6 (0.9-2.9)	p=0.388 OR=1.25 (0.74-2.11) AOR=1.39 (0.80-2.41)
Borrowed to receive treatment	p=0.001 OR=5.96 (2.02-17.54) AOR=5.90 (1.86-18.67)	p=0.376 OR=1.66 (0.54-510) AOR=1.94 (0.571-6.59)	p=0.931 OR=0.94 (0.26-3.31) AOR=0.91 (0.24-3.43)	p=0.094 OR=0.24 (0.04-1.27) AOR=0.086 (0.01-0.718)
Indebted at the time of interview	p=0.000 OR=8.23 (2.59-26.12) AOR=8.85 (2.56-30.50)	p=0.473 OR=1.7 (0.38-8.00) AOR=1.86 (0.38-9.04)	p=0.402 OR=1.73 (0.479-6.244) AOR=1.621 (0.443-5.996)	p=0.278 OR=0.295 (0.32-2.67) AOR=0.307 (0.031-2.98)
Catastrophic expenditure (spent >10% of annual household income)	p=0.003 OR=10.92 (2.30-51.7) AOR=9.16 (1.84-45.5)	p=0.008 OR=17.6 (2.124-145.8) AOR=16.5 (1.95-139.8)	p=0.557 OR=1.71 (0.28-10.4) AOR=1.71 (0.277-10.56)	p=0.156 OR=4.91 (0.54-44.5) AOR=5.19 (0.55-48.61)
Husband's job affected due to delivery	p=0.009 OR=2.1 (1.2-3.6) AOR=2.7 (1.4-5.1)	p=0.457 OR=0.79 (0.43-1.45) AOR=0.76 (0.402-1.440)	p=0.492 OR=1.16 (0.75-1.77) AOR=1.2 (0.77-1.90)	p=0.998 OR=0.99 (0.621-1.60) AOR=0.98 (0.60-1.61)
Income reduced after delivery	p=0.002 OR=2.3 (1.36-3.15) AOR=3.08 (1.70-5.59)	p=0.447 OR=0.78 (0.42-1.45) AOR=0.74 (0.39-1.42)	p=0.235 OR=1.293 (0.85-1.97) AOR=1.41 (0.9-2.23)	p=0.994 OR=1.00 (0.62-1.62) AOR=0.99 (0.60-1.64)
Living conditions have declined after delivery	p=0.006 OR=2.1 (1.2-3.7) AOR=2.8 (1.5-5.3)	p=0.508 OR=0.78 (0.38-1.59) AOR=0.82 (0.39-1.73)	p=0.866 OR=1.04 (0.63-1.70) AOR=1.18 (0.70-1.99)	p=0.003 OR=0.36 (0.18-0.70) AOR=0.36 (0.18-0.72)

Data are p values, crude odds ratios, adjusted odds ratios with 95% confidence intervals. AOR is adjusted against caste and parity. AOR=Adjusted odds ratio; OR=Odds ratio

## DISCUSSION

This study adds to our understanding of the consequences of maternal complications and even uncomplicated delivery on women's lives in a low-resource rural setting. The results suggest that women with severe complications suffer a higher risk of mortality for themselves and for their infants in the first year. They also are at higher risk of suffering depression, find it difficult to resume household work, and suffer economic consequences, including catastrophic expenditure. Even after uncomplicated delivery, a significant proportion of women reported inability to resume work or endured economic consequences. Low survival of mothers with severe complications has been reported in Burkina Faso (8), and a follow-up study of 4,700 women in Mali revealed 15 maternal and 5 late maternal deaths (9).

Complications suffered by women were most frequently related to moderate or severe anaemia and haemorrhage. These conditions are related to anaemia and its consequences and imply the need to address it as a priority during the immediate postpartum period. The exact period of follow-up of women detected to have complications soon after delivery would need to be explored through further research. Given that after the introduction of a national conditional cash-transfer programme a large proportion of women delivered in institutions, it would be feasible to introduce such an intervention in the immediate postpartum period before discharge. The findings of the present study also highlight the need for women undergoing home-delivery to be screened for complications and provided postpartum care beginning in the first week. Subsequently, care should also be provided over the next few months to women who were detected to have complications.

Women with severe complications were significantly more likely to experience perinatal deaths. Results of a study of obstetric causes of perinatal deaths in seven developing countries revealed that hypertensive disorders and spontaneous premature delivery are the most common obstetric causes linked to perinatal death (10). Other studies too have linked obstetric complications to perinatal mortality since the same complication affects both mother and foetus (11-13). The link between maternal morbidity and late neonatal and infant mortality is, however, not well-established. In our sample, women with severe complications had higher risk of late neonatal and infant deaths. This risk was over and above that engendered by low birthweight. Similar findings were reported in a study on hospital deliveries in Burkina Faso (8), wherein infants born to women with obstetric complication were 3.7 times more likely to die than those without complications. The higher rate of infant mortality among women with severe complications is possibly linked to a combination of the mother's inability to breastfeed properly, to take care of the infant, and to seek prompt treatment in the event of illness. With over two-thirds of the women with severe complications suffering from severe anaemia in our study, it is likely that concurrent micronutrient deficiency contributed to vitamin A and zinc deficiency in their infants. These deficiencies are associated with lower immunity and infant mortality (14).

Several women with severe complications reported physical symptoms, such as pain in the arms and legs, weakness, and difficulty with self-care and household work. It is likely that these symptoms were a consequence of severe anaemia, which affected most women in this group. Our earlier ethnographic work in this area has revealed that women with anaemia often express their symptoms as ‘pain in the arms and legs’ and ‘weakness’. Studies in the USA also reported postpartum fatigue to be a significant symptom (15,16). Anaemic women suffer from higher levels of fatigue and lower efficiency (17). In a rural society in which household and farm work is a crucial part of women's routine, continuing difficulty in carrying out their household work for up to a year after delivery carries implications for family well-being and productivity.

Fewer than half of the women with complications sought care outside the home, even up to 12 months after delivery. Of those with severe complications, 11% incurred catastrophic expenditure, and more than 15% were indebted up to eight weeks after delivery. Although India is implementing a cash-transfer scheme that pays Rs 1,400 (~US$ 30) to women delivering in government institutions, the findings of our study suggest that women need financial support to meet their own and their infants' health needs in the postpartum year.

The findings of our study on the prevalence of postnatal depression 6-8 weeks after delivery match those from other studies (18). A study in Goa, India, detected postnatal depression in 23% of mothers 6-8 weeks after delivery (19). The higher rates of depression among women with severe maternal complications were also reported in Burkina Faso (8). It is likely that symptoms of complication and inability to carry out household work contributed to depression. Studies in developed countries have identified postpartum anaemia as a significant risk factor in the development of depression (20,21) and a negative correlation between haemoglobin concentrations and depressive symptoms. A study in South Africa reported that postpartum depression, stress, and cognitive impairment in poor women were related to the existence of iron-deficiency anaemia and that this depression responded to iron therapy (22). These findings have important implications for the management of depression, given that rural mothers lack access to psychiatric care, and there is no specific strategy to deal with the problem in the primary-care setting. We feel that, given its high prevalence, risk factors for postpartum depression in low-resource settings should be further explored, and preventive and coping strategies be identified. Primary-care providers such as nurse-midwives should be trained to detect and manage postpartum depression.

The strength of the present study is that it recruited women from the community during the first postpartum week based on a detailed enquiry by skilled care providers about delivery-related complications and a physical examination they performed at that time. It included women with complications from both institutional and home-deliveries; hence, those who were recruited included the poorest women, those residing in remote villages, and those who had delivered at home.

### Limitations

A limitation of our study is the low rate of follow-up at 12 months mainly due to high rates of out-migration from our field area to neighbouring states. A second limitation is that women of various categories in the subset for detailed study were not randomly selected. While women with uncomplicated deliveries and less-severe complications were recruited over a shorter period after data-collection started, it took many months to recruit a sufficient number of women with severe complications because of their lower prevalence.

### Conclusions

The definitions we used for classifying women as having severe complications are not the same as in the definition of the World Health Organization for ‘near miss’ (23) as we selected women who delivered either at home or facility. Our attempt was to classify women with life-threatening complications as those with ‘severe complications’. We included severe postpartum anaemia in this category because in our study very few women with severe anaemia actually received blood transfusion while it is a life-threatening condition. We also included women with caesarean section for selected ‘maternal’ indications—antepartum haemorrhage, obstructed labour, transverse lie, twin pregnancy—in the category of severe complications because these are life-threatening conditions.

**Table 10.** Self-care and social relationships of women with severe complications, less-severe complications, and normal delivery

Self-care and social consequences	6-8 weeks			12 months		
	Severe (n=84) No. (%)	Less severe (n=162) No. (%)	Uncomplicated (n=184) No. (%)	Severe (n=71) No. (%)	Less severe (n=139) No. (%)	Uncomplicated (n=165) No. (%)
Self-care less than before	18 (21.4)	23 (14.2)	35 (19.0)	12 (16.9)	13 (9.4)	6 (3.6)
Difficulty in starting manual labour	NA	NA	NA	38.9	41.7	20.8
Difficulty in resuming household work	57 (67.9)	89 (54.9)	85 (46.2)	44 (62.0)	50 (36.0)	53 (32.1)
Suffered violence/verbal abuse/ignored	3 (3.6)	7 (4.3)	10 (5.4)	2 (2.8)	3 (2.2)	3 (1.8)
Not resumed sexual relationships because of illness	13 (15.5)	6 (3.7)	8 (4.3)	2 (2.8)	1 (0.7)	0 (0.0)

NA=Not applicable

**Table 11.** Self-reported symptoms at 6-8 weeks and 12 months postpartum among women with severe, less-severe complications, and normal delivery

Perceived symptom	6-8 weeks			12 months		
	Severe (n=84) No. (%)	Less severe (n=162) No. (%)	Uncomplicated (n=184) No. (%)	Severe (n=71) No. (%)	Less severe (n=139) No. (%)	Uncomplicated (n=165) No. (%)
Not feeling well*	18 (26)	19 (15)	20 (12)	7 (9.9)	8 (5.8)	4 (2.4)
Fever	33 (39.3)	39 (24.1)	38 (20.7)	26 (36.6)	30 (21.6)	28 (17.0)
Swelling of body	5 (6.0)	2 (1.2)	2 (1.1)	8 (11.3)	0	2 (1.2)
Weakness	46 (54.8)	62 (38.3)	62 (33.7)	37 (52.1)	52 (37.4)	51 (30.9)
Pain in arms and legs	50 (59.5)	82 (50.6)	80 (43.5)	41 (57.7)	54 (38.8)	53 (32.1)
Excessive bleeding	18 (21.4)	37 (22.8)	13 (7.1)	11 (15.5)	21 (15.1)	11 (6.7)
Cough	14 (16.7)	16 (9.9)	15 (8.2)	11 (15.5)	14 (10.1)	13 (7.9)
Abdominal pain	19 (22.6)	32 (19.8)	30 (16.3)	19 (26.8)	24 (17.3)	27 (16.4)
Vaginal discharge	14 (16.7)	27 (16.7)	25 (13.6)	17 (23.9)	22 (15.8)	26 (15.8)
Burning sensation in urine	6 (7.1)	12 (7.4)	8 (4.3)	6 (8.5)	12 (8.6)	9 (5.5)
Breast engorgement	6 (7.1)	14 (8.6)	10 (5.4)	2 (2.8)	6 (4.3)	1 (0.6)
At least one symptom	59 (70.2)	95 (58.6)	88 (47.8)	47 (66.2)	61 (43.9)	61 (37.0)
Serious illness†	17 (20.2)	21 (13.0)	19 (10.3)	22 (31.0)	22 (15.8)	22 (13.0)

*In response to a general question about how she was feeling, woman responded that she has not been feeling well since delivery

†More than 3 of the following symptoms: fever, swelling, yellowness of body, feels extremely weak, ache in hands and legs, excessive bleeding, breathlessness, cough, and abdominal pain

**Table 12.** Perceptions of health: levels of statistical significance and odds ratios of severe and less-severe complication group compared to those of uncomplicated group at 6-8 weeks and 12 months

Outcome	Compared to women with uncomplicated deliveries			
	Severe complications		Less-severe complications	
	6-8 weeks	12 Months	6-8 weeks	12 months
Not feeling good	p=0.005 OR=0.38 (0.19-0.75) AOR=0.32 (0.15-0.65)	p=0.021 OR=0.23 (0.064-0.80) AOR=0.23 (0.63-0.84)	p=0.349 OR=0.74 (0.39-1.38) AOR=0.70 (0.36-1.36)	p=0.15 OR=0.41 (0.12-1.38) AOR=0.40 (0.11-1.45)
Perceives feverish	p=0.002 OR=2.48 (1.41-4.37) AOR=2.39 (1.30-4.39)	p=0.001 OR=2.83 (1.50-5.31) AOR=2.66 (1.37-5.17)	p=0.445 OR=1.21 (0.73-2.02) AOR=1.26 (0.74-2.15)	p=0.31 OR=1.35 (0.76-2.38) AOR=1.3 (0.72-2.36)
Perceives swelling of body	p=0.039 OR=5.75 (1.09-30.32) AOR=5.55 (1.02-30.0)	p=0.004 OR=10.34 (2.14-50.07) AOR=13.64 (2.55-72.86)	p=0.898 OR=1.13 (0.16-8.17) AOR=1.18 (0.15-9.22)	NA
Feels weakness	p=0.001 OR=2.38 (1.40-4.04) AOR=2.76 (1.57-4.86)	p=0.002 OR=2.43 (1.37-4.30) AOR=2.72 (1.46-5.06)	p=0.376 OR=1.22 (0.79-1.89) AOR=1.24 (0.78-1.97)	p=0.233 OR=1.34 (0.83-2.15) AOR=1.43 (0.87-2.36)
Feels pain in arms and legs	p=0.015 OR=1.91 (1.13-3.22) AOR=2.09 (1.19-3.66)	p=0.000 OR=2.88 (1.63-5.12) AOR=2.93 (1.59-5.41)	p=0.185 OR=1.33 (0.87-2.04) AOR=1.42 (0.91-2.22)	p=0.222 OR=1.34 (0.84-2.15) AOR=1.46 (0.88-2.4)
Excessive bleeding	p=0.001 OR=3.58 (1.66-7.73) AOR=3.93 (1.72-8.95)	p=0.037 OR=2.56 (1.06-6.23) AOR=2.33 (0.94-5.82)	p=0.000 OR=3.89 (1.98-7.62) AOR=4.42 (2.17-8.99)	p=0.020 OR=2.49 (1.16-5.37) AOR=2.57 (1.18-5.64)
Cough	p=0.041 OR=2.25 (1.03-4.91) AOR=3.0 (1.27-7.11)	p=0.081 OR=2.14 (0.91-5.05) AOR=2.99 (1.12-8.02)	p=0.576 OR=1.23 (0.59-2.58) AOR=1.43 (0.65-3.16)	p=0.504 OR=1.31 (0.59-2.89) AOR=1.61 (0.67-3.86)
Abdominal pain	p=0.22 OR=1.5 (0.78-2.85) AOR=1.5 (0.79-3.16)	p=0.067 OR=1.87 (0.96-3.64) AOR=1.81 (0.90-3.64)	p=0.405 OR=1.26 (0.72-2.19) AOR=1.44 (0.81-2.59)	p=0.834 OR=1.07 (0.58-1.95) AOR=1.04 (0.56-1.94)
Vaginal discharge	p=0.51 OR=1.27 (0.62-2.59) AOR=1.38 (0.65-2.92)	p=0.14 OR=1.68 (0.85-3.35) AOR=1.54 (0.72-3.28)	p=0.42 OR=1.27 (0.70-2.29) AOR=1.24 (0.68-2.27)	p=0.99 OR=1.01 (0.54-1.86) AOR=1.02 (0.54-1.96)
Burning sensation in urine	p=0.345 OR=1.69 (0.57-5.04) AOR=1.70 (0.52-5.64)	p=0.39 OR=1.6 (0.55-4.67) AOR=1.27 (0.41-3.97)	p=0.23 OR=1.76 (0.70-4.42) AOR=1.75 (0.68-4.53)	p=0.28 OR=1.637 (0.67-4.01) AOR=1.51 (0.6-3.78)
Breast engorgement	p=0.59 OR=1.33 (0.47-3.81) AOR=0.96 (0.30-3.08)	p=0.21 OR=4.75 (0.42-53.29) AOR=5.06 (0.44-57.73)	p=0.25 OR=1.64 (0.71-3.81) AOR=1.41 (0.59-3.36)	p=0.065 OR=7.4 (0.88-62.22) AOR=6.94 (0.78-61.87)
At least one-symptom illness	p=0.001 OR=2.57 (1.48-4.46) AOR=2.73 (1.52-4.89)	p=0.000 OR=3.34 (1.86-5.99) AOR=3.34 (1.81-6.18)	p=0.045 OR=1.54 (1.01-2.36) AOR=1.70 (1.08-2.66)	p=0.22 OR=1.33 (0.84-2.11) AOR=1.41 (0.87-2.28)
Serious illness*	p=0.359 OR=1.42 (0.66-3.02) AOR=1.96 (0.84-4.59)	p=0.002 OR=2.84 (1.45-5.56) AOR=3.356 (1.59-7.05)	p=0.925 OR=0.96 (0.48-1.94) AOR=1.19 (0.59-2.56)	p=0.51 OR=1.24 (0.65-2.34) AOR=1.34 (0.68-2.65)

*More than 3 of the following symptoms: fever, swelling, yellowness of body, feels extremely weak, ache in hands and legs, excessive bleeding, breathlessness, cough, and abdominal pain. Data are p values, crude odds ratios, and adjusted odds ratios with 95% confidence intervals. AOR is adjusted against caste and parity. AOR=Adjusted odds ratio

**Box. Modified EPDS used for assessment of postpartum depression**

The questions used in the scale included the following:

1. Are you able to laugh and feel happy, if there is something to be happy about? (No=1, Yes=0)
2. Do you enjoy doing things that you enjoyed earlier? (No=1, Yes=0)
3. Do you unnecessarily blame yourself if something goes wrong? (Yes=1, No=0)
4. Do you feel so sad that you keep on crying every now and then? (Yes=1, No=0)
5. Are you able to concentrate on your day-to-day work these days (e.g. if roti gets burnt, too much salt in your food)? (No=1, Yes=0)
6. Do you feel hungry as usual? (No=1, Yes=0)
7. Do you feel tired and weak most of the time and not feel like doing your normal work? (Yes=1, No=0)
8. Do you have difficulty in sleeping at night? (If the baby does not disturb you)? (Yes=1, Cannot sleep because child disturbs=0, No difficulty in sleeping=0)
9. Do you feel sad or miserable most of the time? (Yes=1, No=0)
10. Has the thought of harming yourself occurred to you in recent times? (Yes=1, No=0)

EPDS=Edinburgh Postpartum Depression Scale

Chronic maternal morbidity conditions, especially anaemia over several months and possibly years, affect the ability of women to work and adversely affect their survival, survival of their children, and their economic productivity. If left unaddressed, such conditions will make it more difficult to attain the Millennium Development Goals on maternal mortality, child mortality, and on eradicating extreme poverty and hunger. Till now, maternal health programmes have paid little attention to interventions in the postpartum period. The results of the present study point to a need for health programmes to pay greater attention to maternal health needs not only in the first six weeks but also beyond it, especially for women detected to have complications.

## ACKNOWLEDGEMENTS

The John D. and Catherine T. MacArthur Foundation supported the study. The funder was not involved in the design of the study or conclusions drawn. The views expressed in this paper are solely the responsibility of the authors. The authors thank all the women who participated in the study and acknowledge the invaluable contributions of the nurse-midwives who recruited them. The authors also thank the interviewers (Kailash Kunwar, Pushpa Rawat, and Tajuna Rawat). Finally, they thank Dr. Priti Arun for her help in developing the scale to assess the psychological consequences.
